# Fabrication and Optimization of ChE/ChO/HRP-AuNPs/c-MWCNTs Based Silver Electrode for Determining Total Cholesterol in Serum

**DOI:** 10.1155/2016/1545206

**Published:** 2016-01-18

**Authors:** Kusum Lata, Vikas Dhull, Vikas Hooda

**Affiliations:** ^1^Centre for Biotechnology, Maharshi Dayanand University, Rohtak, Haryana 124001, India; ^2^Department of Bio & Nano Technology, Guru Jambheshwar University of Science & Technology, Hisar, Haryana 125001, India

## Abstract

The developed method used three enzymes comprised of cholesterol esterase, cholesterol oxidase, and peroxidase for fabrication of amperometric biosensor in order to determine total cholesterol in serum samples. Gold nanoparticles (AuNPs) and carboxylated multiwall carbon nanotubes (cMWCNTs) were used to design core of working electrode, having covalently immobilized ChO, ChE, and HRP. Polyacrylamide layer was finally coated on working electrode in order to prevent enzyme leaching. Chemically synthesised Au nanoparticles were subjected to transmission electron microscopy (TEM) for analysing the shape and size of the particles. Working electrode was subjected to FTIR and XRD. The combined action of AuNP and c-MWCNT showed enhancement in electrocatalytic activity at a very low potential of 0.27 V. The pH 7, temperature 40°C, and response time of 20 seconds, respectively, were observed. The biosensor shows a broad linear range from 0.5 mg/dL to 250 mg/dL (0.01 mM–5.83 mM) with minimum detection limit being 0.5 mg/dL (0.01 mM). The biosensor showed reusability of more than 45 times and was stable for 60 days. The biosensor was successfully tested for determining total cholesterol in serum samples amperometrically with no significant interference by serum components.

## 1. Introduction

With increasing mortality rate due to cardiovascular diseases (CVDs) in present scenario, it is necessary to develop more advanced methods for diagnosis. These advanced diagnostic methods should sense the disease at an early stage and prevent it from being fatal. Cholesterol is considered as risk factor when the blood cholesterol is above the normal level and so causes the risk of cardiovascular disease (CVD) [1]. In addition, high blood pressure or diabetes may also increase the risk even more. Elevated level of cholesterol in blood is one of the factors responsible for coronary artery disease, hypertension, nephritic syndrome or cirrhosis, atherosclerosis, heart attack, and stroke [2]. When too much of cholesterol circulates in the blood, it slowly builds up layer inside the inner walls of the arteries which makes them narrow and less flexible [2].

There are several methods such as calorimetric method [3], liquid/gas chromatographic method, HPLC, spectrophotometric methods, and thermistor based assays used to determine cholesterol concentration [4–8]. The above methods are fast, accurate, and sensitive but suffer from limitations: they require trained manpower, they require sample pretreatment, a lot of time is required, and they are only confined to laboratory lacking on-site monitoring. A promising alternate to these methods is a biosensor which is able to determine the cholesterol more rapidly and to a more sensitive level with a plus point of portability [9]. The biosensor assembly comprised three components: biorecognition layer (enzyme in this case) which specifically interacts with analyte and generates the biological response, a transducer which senses these responses and processes the biological response to a measurable signal, and a display unit which is the third component. Transducers are an important part of assembly of biosensor in determining accuracy and sensitivity [10]. Immobilizing ChO on various supports helps in directly determining concentration of cholesterol in different samples. As serum cholesterol is present in the form of ester, in order to determine total cholesterol, ChE is required along with ChO. Cholesterol esterase first hydrolyses the esterified cholesterol and is further oxidized by cholesterol oxidase for producing cholest-4-en-3-one and hydrogen peroxide (H_2_O_2_). In amperometric biosensors electric potential is applied on H_2_O_2_ which get oxidized to produce electrons and the flow of electrons produces current. The current generated is directly proportional to cholesterol concentration in serum:

(1) Cholesterol ester+H2O→Cholesterol EsteraseCholesterol+fatty acidsCholesterol+O2→Cholesterol OxidaseCholest-4-en-3-one+H2O2H2O2→Electric Potential2H++O2+e−

A new approach to reduce interference is the use of horseradish peroxidase (HRP). HRP has redox centre associated with ferroheme/ferriheme pair. Conversion of reduced state to oxidized state is done through direct transfer of electrons. In its 3D configuration HRP has heme group present in outer region which enhances the direct transfer of electrons between redox centre and conducting sites present on transducer [11]. Regeneration of HRP is done via direct electron transfer:

(2) H2O2+2H++HRPRed.⟶2H2O+HRPOxd.HRPOxd.+2e−⟷HRPRed.

Immobilizing enzyme on suitable support may lower the Michaelis constant *K*_*m*_ or it may enhance bimolecular rate constant, which improves the biosensor performance [12–15]. Cholesterol hydrolysing enzymes ChO and ChE have been immobilized on conducting polymers [16] like polypyrrole films [17], PVC Strip [18], PB/polypyrrole (PPy) composite film [19], cellulose acetate/carbon electrode [20], cellulose acetate/Pt electrode [21], electropolymerised PPy films [22], poly 3-thiopheneacetic acid film/Pt electrode [23] and PANI–pTSA-Ag/ITO [11], and sol-gel films [24]. Evolution of nanoscale materials has revolutionised the field of biosensors also. The nanoscale dimensions, their graphitic surface chemistry, and electrocatalytic properties of carbon nanotubes make them an interesting material for sensing purpose. Single and multiwalled carbon nanotubes [25, 26], MWCNTs [27], CNT-chitosan/GCE [28], Fe_3_O_4_-SiO_2_/MWCNT [29], graphene modified graphite electrode [30], graphene/Pt NPs [31], Pt/Au ZnO nanorods [32], NSPANI-AuNP-GR/ITO [33], AuNPs/Au electrode [34], graphite-teflon matrix [35], and Pt-Pd bimetallic nanoparticle decorated grapheme [36] are some of the supports which have been used for the transduction of generated biological signals to electrical signals for cholesterol determination. PVC has also been used to immobilize cholesterol hydrolysing enzymes. ChO and ChE were covalently immobilised on surface of PVC beaker which act as reaction cell and HRP was incorporated in carbon electrode [37].

In the present method, the combination of three enzymes was used for the development of electrochemical biosensor by immobilization of three enzymes (ChO, ChE, and HRP) on AuNPs and c-MWCNT based electrode for determining cholesterol in serum cholesterol.

## 2. Materials and Methods

### 2.1. Chemicals and Reagents

Cholesterol esterase was purified from* Pseudomonas *species (165.8 Units/g); 4-amino-phenazone, Triton X-100, cholesteryl acetate, and carboxylated multiwalled carbon nanotubes (c-MWCNTs) were purchased from Sigma Chemical Co., USA. Cholesterol oxidase from* Streptomyces* sp. (500 units/10 mg) and horseradish peroxidase (HRP) (80 U/mg) were obtained from SISCO Research Laboratory Pvt. Ld., Mumbai. Silver wire was purchased from local market. All other chemicals used during the experimentation were of analytical grade. Gold nanoparticles (AuNPs) were synthesised at Centre for Biotechnology, Maharshi Dayanand University, Rohtak.

### 2.2. Instrumentation

Potentiostat (PSTAT mini 910, Metrohm, Switzerland) was used for all electrochemical studies. Ultrasonication was done with Chrom Tech Ultrasonic Liquid Processor. For TEM JEM-2100F microscope (AIRF, JNU, New Delhi) was used; Varian 7000 FTIR spectrometer (AIRF, JNU, New Delhi) was used for performing Fourier transform infrared (FTIR) spectroscopy. X-ray diffraction (XRD) facility for gold nanoparticles and c-MWCNTs was provided by Department of Physics, M.D. University, Rohtak. Shimadzu corporation UV 2450 spectrophotometer was used for spectrophotometric measurement at Centre for Biotechnology.

### 2.3. Synthesis of Gold Nanoparticles

Chemical synthesis of gold nanoparticles was done using citrate reduction method [38]. 100 mL of 0.001% gold chlorous acid (HAuCl_4_) was boiled at 97°C with continuous stirring using magnetic stirrer. Then, the 1% sodium citrate solution was added; within 4 to 5 min solution turns wine red and was further heated for 4 to 5 min and then cooled down at room temperature. Finally centrifuged pellets were dried for further use. Transmission electron microscopy (TEM) of the synthesised AuNPs was done on commercial basis at AIRF, JNU, New Delhi, for the confirmation of shape of newly synthesised nanoparticles and size range of particles.

### 2.4. Fabrication of ChE/ChO/HRP-AuNPs/c-MWCNT Modified Silver Working Electrode

Fabrication strategy of working electrode comprised mixing c-MWCNTs and gold nanoparticles (AuNPs) in paraffin oil in fixed proportion until a consistent paste is obtained. A plastic hollow tube (3 cm × 4 mm) was filled with the nanomaterial passed obtained above. The silver (Ag) wire was cleaned with ethanol and ddH_2_O by sonication and then inserted in the paste filled tube for achieving the electrical contact. Then the above electrode was allowed to dry and then immersed in a mixture of ChO, ChE, and HRP solution for 2 hrs, so that the enzyme can bind on electrode surface. The carboxyl (COOH) group present on MWCNTs forms amide bond with the amino group present on the enzyme leading to the formation of covalent bond. Then, the electrode surface was covered with a thin film of polyacrylamide (PAA) which helps in preventing enzyme from leaching.

### 2.5. Characterisation of Carbon Based Working Electrode

The nanomaterial based core of working electrode (AuNPs/c-MWCNTs/Ag electrode) and enzyme bound electrode (ChE/ChO/HRP-AuNP/c-MWCNTs/Ag electrode) were characterised using Varian 7000 FTIR spectrometer (at AIRF, JNU, New Delhi); before coating with PAA Fourier transform infrared (FTIR) spectroscopy, sample preparation was done in KBr. X-ray diffraction (XRD) studies were also performed to analyse the stability of c-MWCNTs on mixing with AuNPs. Scanning Electron Microscopy (SEM) was done for analysing the modifications in the surface morphology of the working electrode at different stages of fabrication.

### 2.6. Assembly of Cholesterol Biosensor

An amperometric cholesterol biosensor was assembled using ChE/ChO/HRP-AuNPs/c-MWCNT Ag as enzyme immobilized working electrode, Ag/AgCl pure as reference, and Pt wire as auxiliary electrode connected via Potentiostat.

### 2.7. Electrochemical Study of ChO/ChE/HRP-AuNPs/c-MWCNT Ag Electrode

Cyclic voltammetry (CV) studies, Electrochemical Impedance Spectroscopy (EIS), and all other amperometric detections throughout the experiment were performed on a Potentiostat (PSTAT mini 910, Metrohm) using three-electrode system in electrochemical cell. All of the cyclic voltammetric measurements were performed at room temperature and were continuously recorded from −0.4 to +0.4 V with different scan rates (25, 50, and 100 mV/s).

### 2.8. Kinetic Study of Present Method

Kinetic properties of newly developed method were studied which include optimum pH, temperature, response time, effect of substrate (cholesteryl acetate) concentration, *K*_*m*_, and *I*_max_.

### 2.9. Evaluation of the Present Method

Evaluation of present method was carried out with respect to linearity, minimum detection limit, and percent analytical recovery along with precision and accuracy. The effect of interfering species on performance of biosensor was studied. The storage stability with time and repeatability of present method were also analyzed.

#### 2.9.1. Linear Working Range and Minimum Detection Limit

Linearity range and minimum detection range were calculated by plotting values against the values of standard graph.

#### 2.9.2. Analytical Recovery

Reliability of the method was tested using different concentrations of cholesteryl acetate (100 mg/dL and 200 mg/dL) (2.33 mM and 4.66 mM) by spiking the serum samples and mean analytical recoveries of cholesteryl acetate were determined.

#### 2.9.3. Precision

The reproducibility of the present method and the total cholesterol level was determined in the sample on the same day (within batch) and in the same sample after storage at 4°C for one week (between batches); coefficients of variation (CVs) were calculated for the present method.

#### 2.9.4. Accuracy

For determining accuracy of newly developed method, the 10 serum samples were spiked with cholesteryl acetate and then tested by standard Bayer's enzo kit (*x*) and also by present method (*y*); then the values obtained by both methods were co-related and regression equation was obtained.

#### 2.9.5. Effect of Interfering Substances

The response of the present method was analyzed in the presence of interfering substances found in serum such as pyruvate, glucose, citrate, Ca^2+^, uric acid, ascorbic acid, acetone, urea, and bilirubin. The effect of interference by these substances was determined by adding the interfering species in the reaction mixture one by one at their physiological concentration.

### 2.10. Storage Stability and Reusability of the Present Method

Before every use the enzyme electrode was cleaned using washing buffer (0.01 M phosphate buffer saline, pH 7.2 with 0.1% tween 20). The stability of the working electrode was investigated over a period of 60 days, when stored at 4°C. The response of working electrode was measured once in every 5 days.

### 2.11. Application of the Newly Developed Method

Blood samples (1 mL each) with different age and sex group were collected from healthy persons and persons suffering from disease due to elevated cholesterol level after 12 hours of fasting, at Pt. BDS PGIMS, Rohtak. The blood samples were centrifuged at 1500 ×g for 5 min and resulting supernatant (serum) was collected for determination of cholesterol level. The test for the serum total cholesterol was carried out by the present method. ChO, ChE, and HRP immobilized onto the working electrode surface catalyze the hydrolysis of cholesteryl acetate and produce H_2_O_2_ which is then oxidized by HRP. HRP itself is regenerated as it passes the electron to the electrode directly. Current produced in the process is directly proportional to the concentration of the H_2_O_2_ produced which itself is directly proportional to total cholesterol.

## 3. Results and Discussion

### 3.1. Characterization of Gold Nanoparticles (AuNPs)

**Figure 1:**
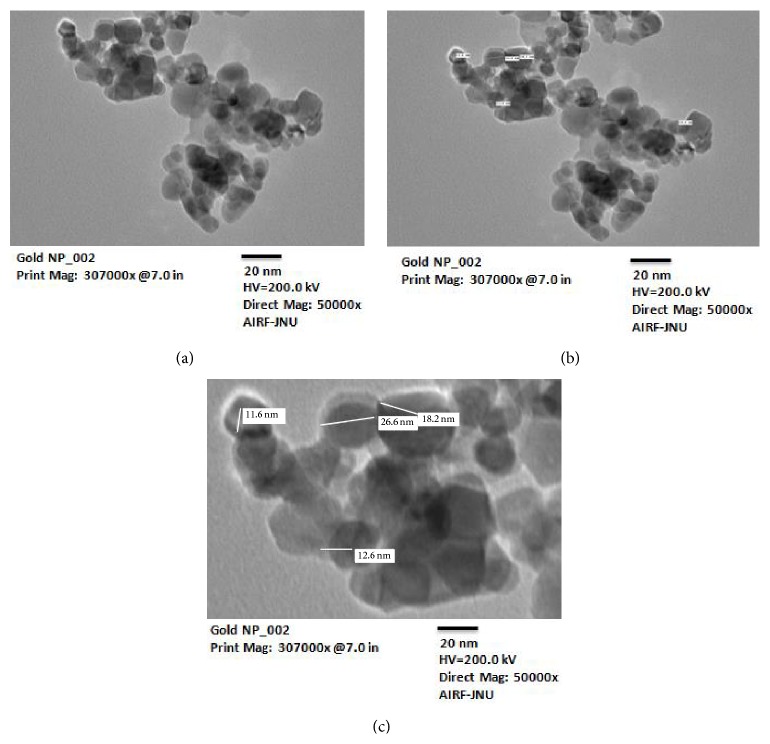
Transmission electron microscopy (TEM) of gold nanoparticles. (a) Aggregates of gold nanoparticles, (b) dimensions of a tuft of nanoparticles, and (c) enlarged image for size analysis.

The lab synthesised AuNPs were characterized by transmission electron microscopic (TEM) study. The data showed that nanoparticles were of spherical shape ranging from 10 to 30 nm in size (Figures 1(a), 1(b), and 1(c)).

### 3.2. Analysis of Gold Nanoparticles Decorated c-MWCNTs by XRD

**Figure 2:**
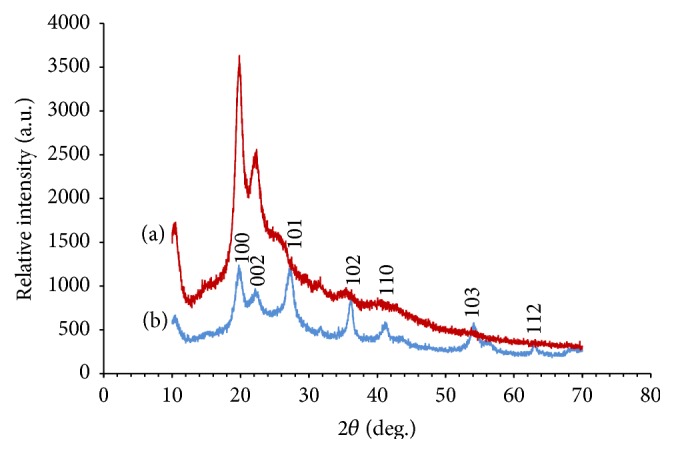
X-ray diffraction patterns of c-MWCNTs (a) and AuNP/c-MWCNTs (b).

Structural stabilization of MWCNTs and working electrode (AuNPs/c-MWCNT) was examined by XRD characterization, as analysed characteristic peaks of c-MWCNTs (Figure 2, Curve (a)) persisted even in the diffraction peaks of AuNPs and c-MWCNT paste (Figure 2, Curve (b)). Peaks appeared at 19.6 and 21.5 which are the unique characteristic peaks of c-MWCNTs which also are present in Curve (b) ensuring that the typical graphitic signature structure of c-MWCNTs is stable even when AuNPs were mixed with c-MWCNT.

### 3.3. Confirmation of Covalent Immobilization by FTIR

FTIR spectra of AuNPs/c-MWCNTs showed absorption peaks near 1011 cm^−1^, 1545 cm^−1^, and 2250 cm^−1^, which are signature peaks of CNTs. After immobilization of enzyme, that is, ChE/ChO/HRP-AuNPs/c-MWCNTs, a new signature peak appeared at 1537 cm^−1^ and 1630 cm^−1^ and a broad peak rose at 3287 cm^−1^ which is due to carbonyl stretch and amide bond.

### 3.4. Surface Morphology of the Newly Fabricated Electrode

**Figure 3:**
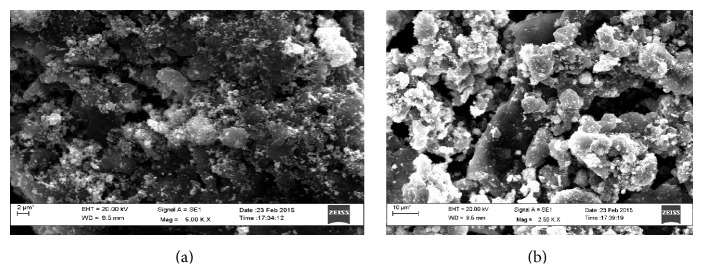
SEM images of (a) electrode without enzyme and (b) with enzyme.

Surface morphology of the working electrode was analysed using Scanning Electron Microscopy at the different stages of fabrication. A visual change had been noticed in the morphological characteristic of the electrode surface with the deposition of material. The SEM images of working electrode without and with enzyme have been shown in Figure 3.

### 3.5. Electrochemical Analysis of ChE/ChO/HRP-AuNPs/c-MWCNT Ag Electrode

**Figure 4:**
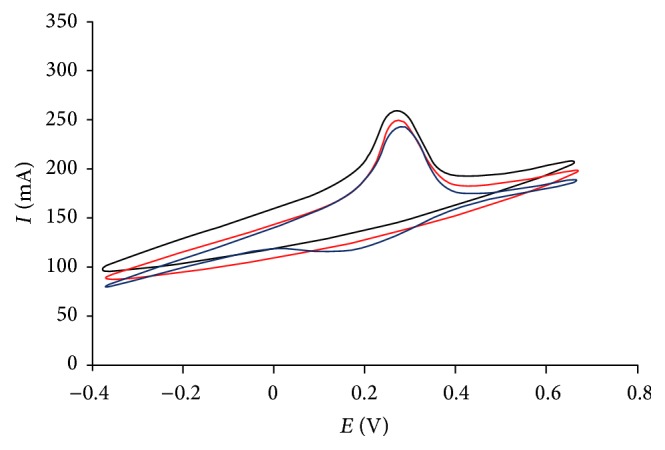
Cyclic voltammogram of ChE/ChO/HRP-AuNPs/c-MWCNTs Ag electrode at various scan rates.

**Figure 5:**
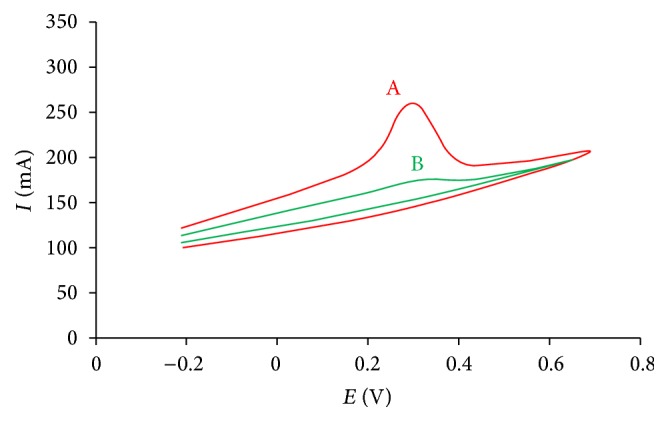
Cyclic voltammogram. (A) ChE/ChO/HRP-AuNPs/c-MWCNTs; (B) bare silver electrode.

The electrochemical study of working electrode was carried out by using cyclic voltammetry. For determination of reduction potential of HRP immobilized on electrode core, it was immersed in 0.01 M H_2_O_2_ for 30 min and then washed with distilled water before performing cyclic voltammetry. Cyclic voltammetry of ChE/ChO/HRP-AuNPs/c-MWCNT Ag electrode was reported from −0.4 and +0.8 V at particular scanned rates of 25 mV/s, 50 mV/s, and 100 mV/s (Figure 4). An optimum scan rate of 50 mV/s was perceived for the above fabricated biosensor. The reproducibility of the fabricated biosensor was examined by running four CV cycles at scan rate of 50 mV/s (Figure 5). During the entire examination, a sharp peak at 0.27 V was observed; this arises as a result of oxidation of HRP present on electrode. So, it was estimated that ChE/ChO/HRP-AuNPs/MWCNT Ag electrode offered ultimate signal and least noise at 0.27 V and was further employed to analytical determinations. The biosensor works at lower potential in comparison to other available biosensors which is due to the large surface area of gold nanoparticles and their highly conductive nature.

### 3.6. Kinetic Study of the Present Method

To improve the working performance of the biosensor various parameters such as working potential, temperature, time, and concentration of substrate and pH value on the fabricated biosensor were analyzed.

#### 3.6.1. Response for Working Potential

**Figure 6:**
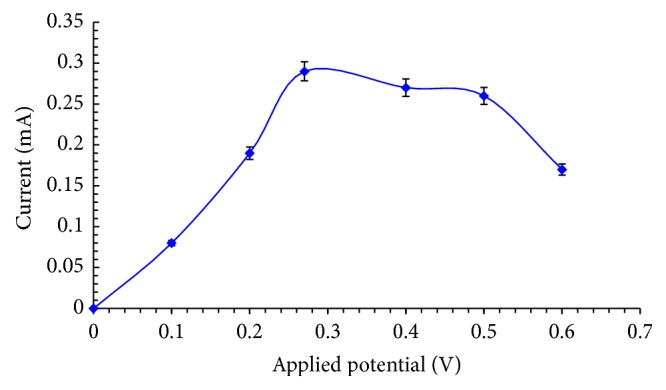
Response of current with applied potential.

The response of current with varying applied potential on the biosensor had been shown in Figure 6. The working potential was skipped from 0.0 V to 0.7 V. With the increase in working potential, increase in steady-state current response was observed. Firstly, it showed a significant increase in current value from +0.1 V to +0.27 V and then reached a level from +0.27 V to +0.5 V and declined slightly after +0.5 V. Therefore, +0.27 V was selected as the working potential for detection of cholesterol by the biosensor.

#### 3.6.2. Response for pH

**Figure 7:**
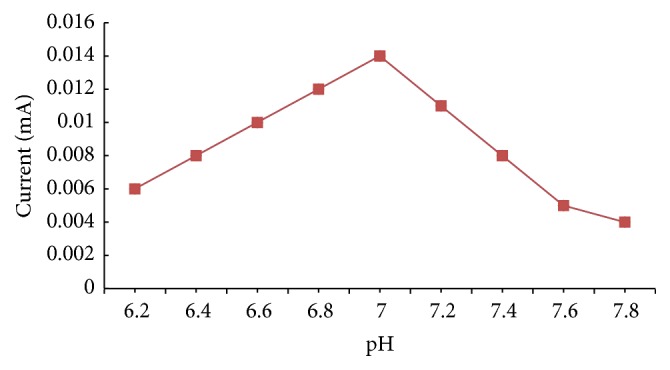
Effect of pH on current response of the present method.

The pH of reaction buffer was varied in the pH range, pH 4.5 to 8.0, to find the optimum pH using sodium succinate buffer (pH 4.5–5.5) and sodium phosphate buffer (pH 6.0–8.0) at a final concentration of 0.02 M. All other variants were of standard assay condition except pH. Optimum pH was found to be 7 for the biosensor (Figure 7).

#### 3.6.3. Response for Temperature

**Figure 8:**
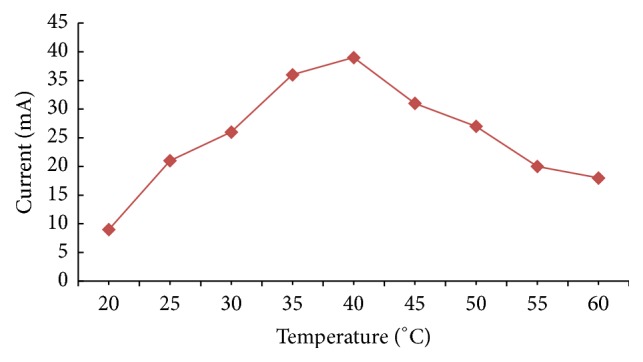
Effect of incubation temperature on the response of present method.

The response of biosensor for continuous raise of temperature by 5°C from 20°C to 60°C was interrogated. It was found that the biosensor showed maximum response at 40°C (Figure 8). The microenvironment provided by support used for immobilization makes it thermally stable and maintains its biological activity.

#### 3.6.4. Response with Time

**Figure 9:**
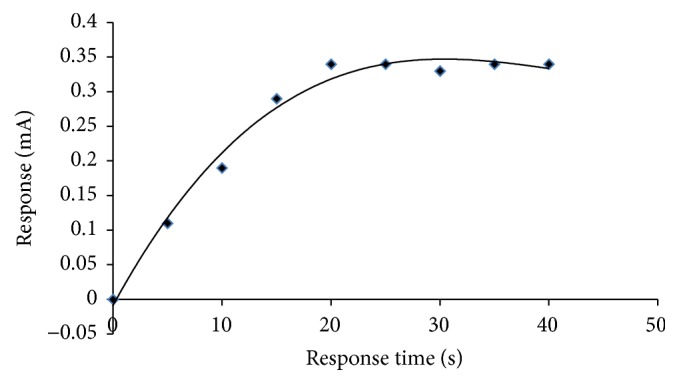
Response time of the current method.

The amperometric response was measured from 5 s to 60 s at interval of 10 s. The response time increases from 5 to 20 s and later attains stability (Figure 9).

#### 3.6.5. Response for Substrate Concentration

**Figure 10:**
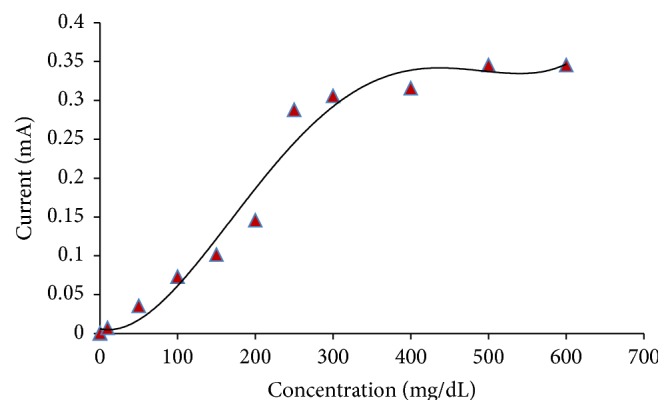
Effect of cholesteryl acetate concentration on the present method.

A linear relationship among the substrate concentration from 0.5 mg/dL to 250 mg/dL and current was observed. The current approach gave a hyperbolic curve between current response and cholesteryl acetate concentration. A significant response was observed up to a concentration of 500 mg/dL (Figure 10).

#### 3.6.6. Resolving *K*_*m*_ and *I*_max_ Values

**Figure 11:**
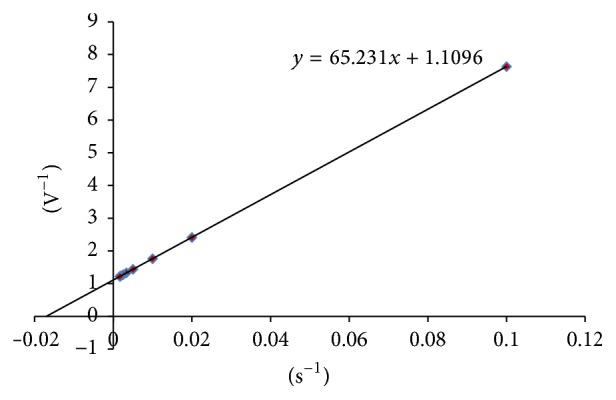
Lineweaver-Burk plot of the present method.

The *K*_*m*_ (app) and *I*_max_ (app) values were resolved by estimating the slope and intercept for the reciprocal plot of current versus cholesteryl acetate concentrations, that is, double reciprocal plot or Lineweaver-Burk plot. The *K*_*m*_ and *I*_max_ values obtained were 58.7 mgdL^−1^ and 0.9 mAs^−1^, respectively (Figure 11).

### 3.7. Assessment of the Current Method

#### 3.7.1. Linear Range for Working and Minimum Limit of Detection

Both the linear range for working and minimum limit of detection of a biosensor are considered while interrogating the performance of a biosensor. In the current method, the standard graph between substrate concentration and current response was used for estimation of linear range for working and minimum limit of detection. The linear range for working offered by current biosensor is 0.5 mg/dL–250 mg/dL and minimum limit of detection limit is 0.5 mg/dL which is far better than previous announced biosensors (Table 1).

#### 3.7.2. Analytical Recovery

By analytical recovery of added enzyme cholesteryl acetate reliability of the current biosensor was calculated (Table 1). The mean analytical recoveries for 100 mg/dL and 200 mg/dL of added cholesteryl acetate were 99.1% and 98.6%, respectively.

#### 3.7.3. Precision

The concentration of total cholesterol was calculated on the same day (within batch) and in the same sample after storage at 4°C for one week (between batches) in the serum sample repeatedly to examine the reproducible nature of the current biosensor. The values for coefficients of variation (CVs) were < 0.61% and < 0.98% for within batch and between batches, respectively (Table 2). The results were far better than various earlier reported methods (Table 6).

#### 3.7.4. Accuracy

**Figure 12:**
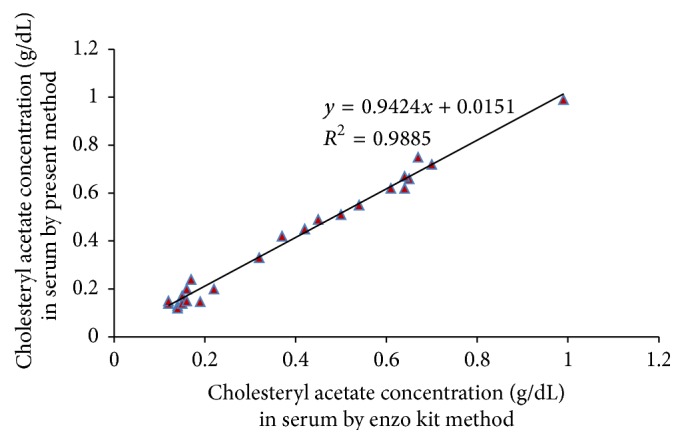
Correlation between cholesteryl acetate concentrations in serum determined by standard enzo kit method (*x*-axis) and by the present method (*y*-axis).

The level of cholesteryl acetate added to serum samples was computed by using the standard method, that is, enzo kit (*x*) and current method (*y*), and accuracy of the current biosensor was analysed (Figure 12). The regression equation *y* = 0.9424*x* + 0.0151 was used to attain cholesteryl acetate results for newly fabricated working electrode with a good correlation (*R* ^2^ = 0.988) as compared to standard method. All these results showed that the current biosensor offers excellent accuracy.

#### 3.7.5. Interference Study

Among the various serum substances investigated for possible interference on the response of the present method, none caused any significant interference on the performance of cholesterol biosensor. Effect of different substances on the working of biosensor has been shown in Table 3.

### 3.8. Reliability and Stability

**Figure 13:**
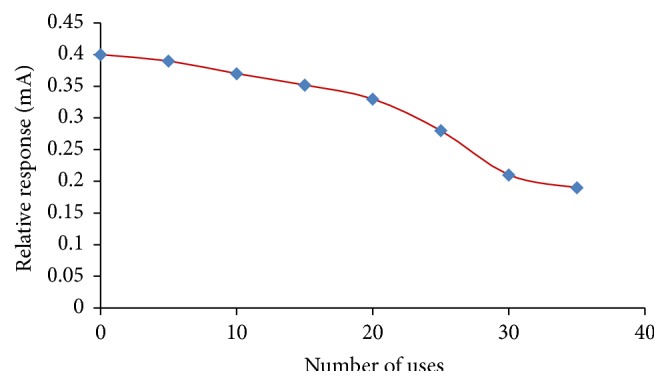
Reusability of the present method.

**Figure 14:**
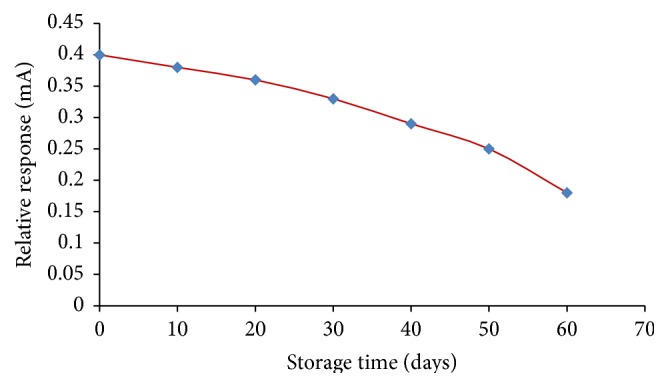
Storage stability of the present method.

The biosensor was reliable and stable. When stored at 4°C, the sensor was stable up to one month and after 35 uses in second month its performance was reduced (Figure 13). Only 45% activity of the biosensor remained at the end of the 2nd month (Figure 14). In comparison to the reported biosensors it showed better stability (Table 6). The immobilization of ChO, ChE, and HRP on AuNPs/c-MWCNTs Ag based working electrode was credited by good stability, reliability, and reproducibility. Further enzyme degrading and leaching was prevented by coating the biosensor with polyacrylamide which also increases the stability of biosensor.

### 3.9. Application of the Newly Developed Method

Concentration of cholesterol was determined in different samples by the newly developed biosensor.  Table 4 represents the total cholesterol in serum of probably healthy individuals including males and females of different age group computed by the current biosensor. The total cholesterol level was found between 154.17 and 225.89 mg/dL for males and 144.56 and 225.58 mg/dL for females which is in normal range.  Table 5 outlines the different working parameters of freshly fabricated biosensor.

## 4. Conclusion

A fresh biosensor was fabricated exploiting the conductive properties of Au nanoparticles and c-MWCNT paste. Covalent immobilisation of ChO, ChE, and HRP on the working electrode was insured by FTIR. This ChE/ChO/HRP-AuNPs/c-MWCNTs modified Ag electrode exhibits enhanced sensitivity in a linear range of 0.5 mg/dL–250 mg/dL (0.01 mM–5.83 mM), quick response time (<20 s), low limit of detection (0.5 mg/dL) (0.01 mM), reproducibility of more than 55 times, and stability of 2 months. A good correlation (*R* ^2^ = 0.988) was obtained with that of standard method. Further, the working electrode was coated with polyacrylamide polymer which provides long time stability and high reusability to the biosensor. The work contributed a competent amperometric approach for detection of total cholesterol in serum.

## Figures and Tables

**Table 1:** Analytical recovery calculated by using added cholesteryl acetate in serum sample.

Cholesteryl acetate added (mg/dL)	Cholesteryl acetate found (mg/dL) (mean (*n* = 5))	% recovery	S.D.
Nil	173.74	—	—
100	271.33	99.11	0.88
200	368.79	98.67	0.93

**Table 2:** Within batch and between batches coefficients of variation for determination of total cholesterol in serum samples.

*n*	Total cholesterol (mg/dL) (mean ± S.D.)	CV (%)
Within batch (6)	170.02 ± 1.04	0.61
Between batches (6)	169.96 ± 1.6	0.98

**Table 3:** Effect of different serum substances on the working of CH biosensor.

Compounds added	Final conc. (physiological conc.) (g/L)	% relative response
None	—	100
Glucose	0.90	99
Uric acid	0.03	100
Ascorbic acid	<17	101
Urea	0.10	98
Ca^2+^	11.5	99
Acetone	0.02	98
Bilirubin	2.2	100

**Table 4:** Total cholesterol level in serum of probably healthy individuals calculated by current biosensor.

Age group (*n* = 08)	Sex	Total cholesterol in serum mg/dL (mean ± S.D.)
<10	M	154.17 ± 2.04
	F	144.56 ± 3.05

11–20	M	170.14 ± 6.57
	F	163.95 ± 4.31

21–30	M	189.24 ± 5.45
	F	185.64 ± 6.08

31–40	M	199.87 ± 7.01
	F	190.02 ± 9.02

41–50	M	208.56 ± 8.18
	F	195.72 ± 8.16

51–60	M	223.56 ± 6.02
	F	217.34 ± 5.06

61 & above	M	225.89 ± 6.05
	F	225.58 ± 9.03

**Table 5:** Working parameters of the newly developed method.

Parameters	Present method
pH	7
Temperature (°C)	40
Working potential (V)	+0.27
*K*_*m*_ (app) (mg/dL)	58.7 (1.36 mM)
*I*_max_ (app) (mA)	0.9
Detection limit (mg/dL)	0.5 (0.01 mM)
Linearity (mg/dL)	0.5–250 (0.01 mM–5.8 mM)
Response time (sec)	20
Storage stability (days)	60

**Table 6:** Comparison of the present method with previously reported biosensor for total cholesterol determination.

Transducer	Method of enzyme immobilization	Working potential	Response time	Detection limit	Linearity	Storage stability	Reference
Laponite clay nanoparticles-pol((12-pyrrol-1-dodecyl)triethylammonium tetrafluoroborate)/Pt disk electrode	ChO, ChE enzyme Entrapment	0.53 V versus Ag/AgCl	50 sec	20 *μ*M	—	20 days	[39]

Screen printed graphite electrode	ChO, ChE, HRP, K4Fe(CN)6 Physical adsorption	−0.2 V versus Ag/AgCl	—	2.81 mM	2.81–13 mM	—	[40]

Polydiaminonaphthalene/Pt disk	ChO, ChE Entrapment	0.7 V versus Ag/AgCl	15 sec	97 *μ*M	Up to 0.8 mM	—	[41]

MWCN/screen printed carbon electrode	ChO, ChE, HRP, K4Fe(CN)6 Physical adsorption	0.3 V versus Ag/AgCl	180 sec	100 mg/dL	100–400 mg/dL	2 months	[27]

3-Aminopropyl-modified controlled-pore glass(APCEG)/rotating disk	ChO, ChE, HRP Covalent cross-linking via Glutaraldehyde	−0.15 V versus Ag/AgCl with TBC as mediator	—	11.9 nM	1.2 *μ*M–1 mM	25 days	[42]

PANI/ITO	ChO, ChE Covalent cross-linking via Glutaraldehyde	0.5 V versus Ag/AgCl	40 sec	50 mg/dL	50–500 mg/dL	6 weeks	[43]

HRP incorporated carbon paste	ChO, ChE Covalent cross-linking on PVC beaker	−0.5 V versus Ag/AgCl	20 sec	2.5 mg/dL	50–550 mg/dL	100 days	[38]

Nanoporous Au networks directly grown on a titanium substrate	ChO, ChE, HRP Physical adsorption Chitosan used as glue	Cyclic voltammetry	—	0.5 mg/dL	0.97–7.8 mM	60 days	[44]

ZnO–CuO composite matrix grown onto ITO coated corning glass	ChO, ChE Physical adsorption	Cyclic voltammetry	5 sec	0.5 mM	0.5–12 mM	—	[45]

c-MWCNT/AuNP	ChO, ChE, HRP Covalent cross-linking via c-MWCNT	0.27 V versus Ag/AgCl	20 sec	0.5 mg/dL	0.5–300 mg/dL	60 days	This work
